# Identification of Quantitative Trait Loci Associated with Plant Adaptation Traits Using Nested Association Mapping Population

**DOI:** 10.3390/plants13182623

**Published:** 2024-09-20

**Authors:** Akerke Amalova, Adylkhan Babkenov, Charlie Philp, Simon Griffiths, Saule Abugalieva, Yerlan Turuspekov

**Affiliations:** 1 Institute of Plant Biology and Biotechnology, Almaty 050040, Kazakhstan; akerke.amalova@gmail.com (A.A.); absaule@yahoo.com (S.A.); 2 Alexandr Barayev Scientific-Production Center for Grain Farming, Shortandy 021600, Kazakhstan; babkenov64@mail.ru; 3 John Innes Centre, Norwich NR4 7UH, UK; charlie.philp@jic.ac.uk (C.P.); simon.griffiths@jic.ac.uk (S.G.); 4 Faculty of Biology and Biotechnology, Al-Farabi Kazakh National University, Almaty 050040, Kazakhstan

**Keywords:** bread wheat, nested association mapping, genome-wide association studies, plant adaptation-related traits

## Abstract

This study evaluated 290 recombinant inbred lines (RILs) of the nested association mapping (NAM) population from the UK. The population derived from 24 families, where a common parent was “Paragon,” one of the UK’s spring wheat cultivar standards. All genotypes were tested in two regions of Kazakhstan at the Kazakh Research Institute of Agriculture and Plant Industry (KRIAPI, Almaty region, Southeast Kazakhstan, 2019–2022 years) and Alexandr Barayev Scientific-Production Center for Grain Farming (SPCGF, Shortandy, Akmola region, Northern Kazakhstan, 2019–2022 years). The studied traits consisted of plant adaptation-related traits, including heading date (HD, days), seed maturation date (SMD, days), plant height (PH, cm), and peduncle length (PL, cm). In addition, the yield per m^2^ was analyzed in both regions. Based on a field evaluation of the population in northern and southeastern Kazakhstan and using 10,448 polymorphic SNP (single-nucleotide polymorphism) markers, the genome-wide association study (GWAS) allowed for detecting 74 QTLs in four studied agronomic traits (HD, SMD, PH, and PL). The literature survey suggested that 16 of the 74 QTLs identified in our study had also been detected in previous QTL mapping studies and GWASs for all studied traits. The results will be used for further studies related to the adaptation and productivity of wheat in breeding projects for higher grain productivity.

## 1. Introduction

Bread wheat is one of the most important agricultural commodities in the world market [1]. In the 2023/2024 year, the global production volume of wheat amounted to almost 785 million tons [2]. In order to continue providing the world’s population with enough wheat in 2050, its yield should be increased by 60% [3]. Therefore, constant productivity and quality improvement are essential for wheat breeding [4]. Wheat cultivation worldwide requires cultivars to adapt to various environmental and climatic conditions. This adaptability is achieved through variations in phenology and traits related to plant architecture [5]. Key phenological and agronomic traits such as heading/flowering time, plant height, and seed maturity time are crucial for adaptation and maximizing yield potential and stability. Identifying the genes underlying this variation and understanding how they interact and perform in different environments are crucial for improving wheat adaptability and optimizing yield potential [6]. 

Phenology plays a crucial role in crop adaptation to specific environments [7]. The perception of the genetic control of phenological traits is essential for breeders to develop cultivars better suited to their local environments. The major genes affecting wheat adaptation include those associated with phenology and plant architecture, such as vernalization (*Vrn*) [8,9,10], photoperiod (*Ppd*) [11,12,13], earliness per se (*Eps*) [14,15,16], and reduced height (*Rht*), in addition to other minor-effect loci. The interaction of these genes determines a genotype’s adaptation to specific environments [13]. Therefore, developing new competitive high-yielding cultivars and adapting them to different environments are key priorities in wheat breeding projects. 

Plant height is a complex trait influenced by various genetic and environmental factors. One of the key genetic factors affecting plant height is the presence of genes related to gibberellin biosynthesis and response, commonly known as the “Green Revolution” genes or reduced height (*Rht*) genes [17]. *Rht* genes are associated with the semi-dwarf phenotype observed in many modern cereal varieties. Semi-dwarf plants have shorter stems compared to their wild-type counterparts, which helps reduce lodging (stem bending or breaking) and allows for a more efficient allocation of resources to reproductive structures, ultimately increasing grain yield [18]. In wheat, the *Rht* genes were first identified in the 1960s. The two main *Rht* genes in wheat are *Rht-B1* and *Rht-D1*, located on the short arms of chromosomes 4B and 4D, respectively. Mutations in these genes reduce stem elongation and contribute to the semi-dwarf phenotype [19].

Plant adaptation and related traits are complex traits controlled by multiple genes [20]. The study of complex quantitative traits in cereals used two common methods: quantitative trait loci (QTL) [21,22] and mapping genome-wide association studies (GWASs) [23,24,25,26,27,28,29,30]. Particularly, with the availability of large-scale genomic resources, the GWAS has emerged as an addition to QTL mapping for complex traits [31]. The GWAS analyzes genetically diverse lines that harbor numerous historical and hereditary recombination events. Additionally, utilizing diverse germplasm in GWASs can potentially capture superior alleles overlooked by conventional breeding practices [31]. 

One of the ways to combine the strength of biparental and association mapping is to employ nested association mapping (NAM) populations [32,33]. NAM populations offer several advantages, including high allelic diversity, high mapping resolution, and low sensitivity to population structure. NAM population is usually developed by using many related progeny within multiple biparental mapping populations, which are developed by selecting a diverse set of founder lines and crossing them to a common reference parent [34]. Founder lines are carefully selected for genetic diversity, allowing them to encompass a wide range of genetic backgrounds. Consequently, these lines may include exotic germplasm, wild relatives, and landraces. The resulting F1 progeny undergo at least four generations of selfing to produce recombinant inbred lines (RILs), whose genomes are mosaics of the parental genomes [34]. This process of shuffling the parental genomes breaks down population structure, introduces recent recombinations, and creates new allele combinations [35]. Consequently, it aids in detecting small effects of QTL and rare alleles from specific parents [35].

Multiparent populations have advantages over biparental as they produce additional recombination breakpoints and increase QTL detection’s allelic diversity and power [36]. Currently, NAM and multiparent advanced generation inter-cross (MAGIC) showed their high potential, for instance, in studies in wheat [37], barley [38,39], durum [40], rice [41], maize [34], sorghum [42], soybean [43], etc. Additionally, QTL studies of traits such as the grain quality of wheat [44,45], grain protein content [46], yield and its components [47], stay-green [48], nitrogen-deficiency tolerance [49], drought tolerance [50], and disease resistance [51,52] in wheat were mainly based on the use of an NAM population. Also, considering genotype–environment interaction patterns suggests a strong influence of the growth environment on the detection of QTLs for plant adaptation [53,54]. As environmental conditions may greatly impact the timing of the heading date and seed maturation, they may also significantly alter yield [55,56]. In Kazakhstan, analogous studies using NAM for cereals, including for wheat, have not been conducted so far. The present study aims to identify quantitative trait nucleotides (QTNs) associated with plant adaptation traits: heading date, seed maturation date, plant height, and peduncle length in the NAM population in the northern and southeastern regions of Kazakhstan through the GWAS.

## 2. Materials and Methods

### 2.1. Plant Materials

The nested association mapping (NAM) population consisted of 290 spring wheat recombinant inbred lines (RILs) derived from 24 families using a single-seed descent method in greenhouse conditions by the John Innes Centre (Norwich, UK). Paragon, a standard UK spring wheat cultivar, was the common parent utilized in the NAM population. The spring wheat NAM panel comprises twenty-four accessions selected as second parental lines, which include (1) 19 landraces sourced from the A.E. Watkins collection, (2) 2 lines from CIMMYT Core Germplasm (CIMCOG), and (3) 2 cultivars: Baj and Wylakatchem (Table 1) [57,58]. 

### 2.2. Evaluation of the Nested Association Mapping Population for Variation in Studied Traits

The studied plants of the NAM population were tested in the field of two regions of Kazakhstan: (1) at the Kazakh Research Institute of Agriculture and Plant Industry (KRIAPI, Almaty region, Southeast Kazakhstan, 2019–2022 years) and (2) Alexandr Barayev Scientific-Production Center for Grain Farming (SPCGF, Shortandy, Akmola region, Northern Kazakhstan, 2019–2022 years). All genotypes and two local standards (check cultivars) “Kazakhstanskaya 4” in KRIAPI and “Astana” in SPCGF were planted in both locations with two replications in a one-meter plot using a randomized complete block design. The distance between rows was 15 cm, with a 5 cm distance between plants [59]. The table presented in Table 2 displays the meteorological conditions recorded during the trials. The studied traits consisted of plant adaptation-related traits, including heading date (HD, days), seed maturation date (SMD, days), plant height (PH, cm), and peduncle length (PL, cm). HD was recorded as the number of days from emergence to the day when half of the spikes appeared in 50% of the plants. SMD was measured as the number of days between heading days and maturation days. PH was measured at harvest maturity, when the maximum height was achieved, from the ground level to the top of the spikes (excluding awns). PL was measured as the length of the first peduncle. Each one-meter plot consisted of seven rows, and three randomly selected plants per row were analyzed for PH and PL. We studied 21 plants per each of 290 genotypes per replication. A similar approach was taken for the second replication. The mean for two replications was calculated using averages in each replication. In addition, the yield per m^2^ (YM2, g/m^2^) was analyzed in both regions.

### 2.3. Genotyping, Population Structure, and Genome-Wide Association Studies

The studied collection was genotyped using the Axiom Wheat Breeder’s Genotyping Array with 35K single-nucleotide polymorphisms (SNPs) [60]. In total, 10,448 polymorphic SNP markers were used in the GWAS after filtering missing data (≥50%) and the minor allele frequency (MAF) ≥ 5%. [61]. Estimation of the linkage disequilibrium (LD) for each chromosome in the 290 RILs of the NAM population was performed in TASSEL version 5.0, and it was estimated and visualized at r^2^ = 0.1 using the R packages version 4.3.0. The association mapping was conducted using a multivariate linear mixed model (MLMM) in the Genome Association and Prediction Integrated Tool (GAPIT version 3) [62]. MLMM was selected for its balanced integration of fixed and random effects and its ability to detect multiple loci while effectively accounting for population structure and kinship, making it particularly suitable for analyzing the complex traits targeted in this study. The population structure was analyzed using a model-based clustering method using STRUCTURE version 2.3.4 [63,64]. Manhattan plots and SNP density plots were generated using the rMVP package (https://cran.r-project.org/web/packages/rMVP/index.html, accessed on 30 May 2024) [65]. The BLAST tool available on Ensembl Plants for the reference genome of *T. aestivum* (https://plants.ensembl.org/Triticum_aestivum/Tools/Blast, accessed on 25 June 2024) [66] was used to identify the protein-coding genes overlapping with the identified significant QTLs. The analysis of variance (ANOVA), principal component analysis (PCA), and correlation analysis were performed using Rstudio software version 4.3.0 (POSIT, Boston, MA, USA) [67]. The broad-sense heritability index (h_2_^b^), indicating the proportion of phenotypic variation due to genetic factors, was calculated based on the ANOVA results according to Genievskaya et al. [68].

## 3. Results 

### 3.1. Phenotypic Variation of 290 RIL NAM Population for Studied Traits

**Figure 1:**
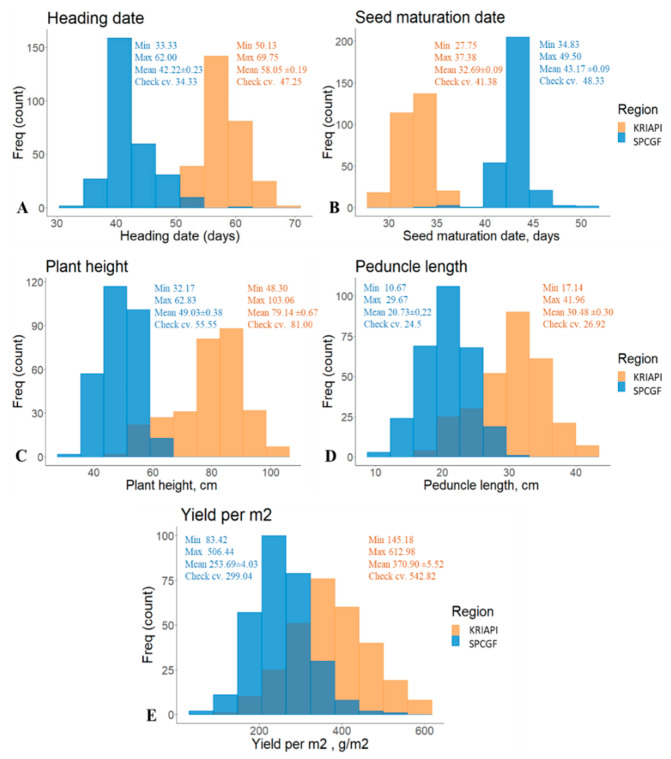
The distributions of four traits: heading date (**A**), seed maturation date (**B**), plant height (**C**), peduncle length (**D**), and yield per m^2^ (**E**) averaged data in the nested association mapping population in the two regions.

The phenotypic assessment of 290 RILs in 24 NAM families was analyzed in seven environments (year-by-location) at the KRIAPI (2019–2022) and SPCGF (2020–2022) (Table S1 and Table 3). The phenotypic variability of four traits between the two regions, including the mean HD, ranged from 42.22 ± 0.23 days at the SPCGF to 58.05 ± 0.19 days at the KRIAPI (Figure 1). The mean PH valued from 49.03 ± 0.38 cm at SPCGF to 79.14 ± 0.67 cm at the KRIAPI (Figure 1), which showed that the mean PH was 30 cm taller in the Almaty region (KRIAPI, south). The average value of YM2 ranged from 253.69 ± 4.03 g/m^2^ (KRIAPI) to 370.90 ± 5.52 g/m^2^ (SPCGF). In general, the *t*-test suggested that the average values of HD, SMD, PH, and PL in the two contrasted regions (KRIAPI and SPCGF) were significantly different (*p* < 0.0001). 

The field performance of PH showed that in the two regions, the mean of the NAM population was shorter than that of the local standard (check cultivars) “Astana” and “Kazakhstanskaya 4” (Figure 1). In contrast, the average HD was from 8 (north) to 11 (southeast) days longer than that of the check cultivars (local standard). Similarly, the average SMD was from 5 (southeast) to 5 (north) days longer than those of the check cultivars (Figure 1). The assessment of the mean YM2 revealed that the yield at the southeastern station was 2.4 times higher than that at the southern station. A total of 11 and 60 accessions showed higher values than check cultivars at the KRIAPI and SPCGF, respectively. 

In general, the *t*-test suggested that the average values of HD, SMD, PH, and PL in the two contrasted regions were significantly different (*p* < 0.0001) (Table S2). 

ANOVA was performed using field data collected from 280 RILs across two locations: KRIAPI (2021–2022) and SPCGF (2020). The ANOVA showed a highly significant difference between the two factors (genotype, environment) for all four studied phenotypic traits. The index of heritability (h_b_^2^) was analyzed for all traits (Table 4), and the highest h_b_^2^ value was noted for HD (29.8%). 

Pearson’s correlation of the average phenotypic values in the two regions suggested different associations. In the southeast region (KRIAPI), the YM2 was negatively correlated with DH and positively correlated with SMD, PH, and PL (Table 5). In the northern region (SPCGF), none of the average data over three years (2020–2022) correlated with YM2. However, when the correlation was analyzed for each year, it was visible that YM2 positively correlated with PH in two out of three years of data (Table 5). At the same time, YM2 was negatively correlated with HD in 2020 and positively correlated in 2022 (Table 5). This controversial correlation was most probably affected by annual rainfall in these years (Table 2).

**Figure 2:**
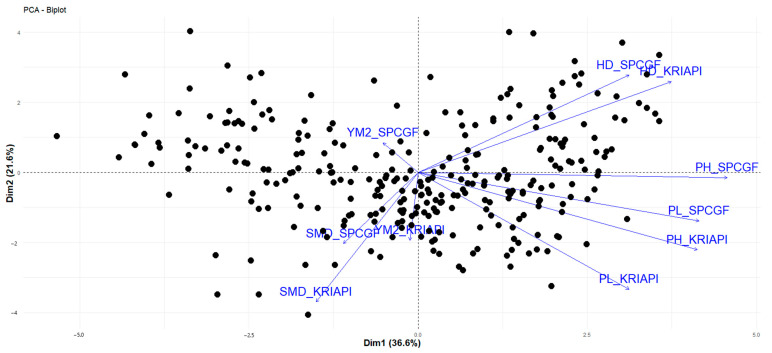
Principal component analysis for studied traits in two regions. Note: RILs—back color, directions of traits—blue color. HD, days—heading date; SMD, days—seed maturation date; PH, cm—plant height; PL, days—peduncle length; YM2, g/m^2^—yield per m^2^; KRIAPI—Kazakh Research Institute of Agriculture and Plant Industry; SPCGF—Alexandr Barayev Scientific-Production Center for Grain Farming.

The PCA for the studied traits showed a relationship of 290 RILs of the NAM population using PC1 and PC2, which explain 36.6% and 21.6% of the total variation, respectively. The results of the PCA of traits are similar to those of the Pearson correlation analysis (Table 5). A similar negative correlation was also noted between HD and SMD, with arrows pointing in different directions (Figure 2). The same trend of negative correlation in terms of yield components was revealed between YM2 and HD at KRIAPI. It is obvious from Figure 2 that the correlation indices were positive for two traits (PH, PL). 

### 3.2. SNP Genotyping and Population Structure of the NAM Population

**Figure 3:**
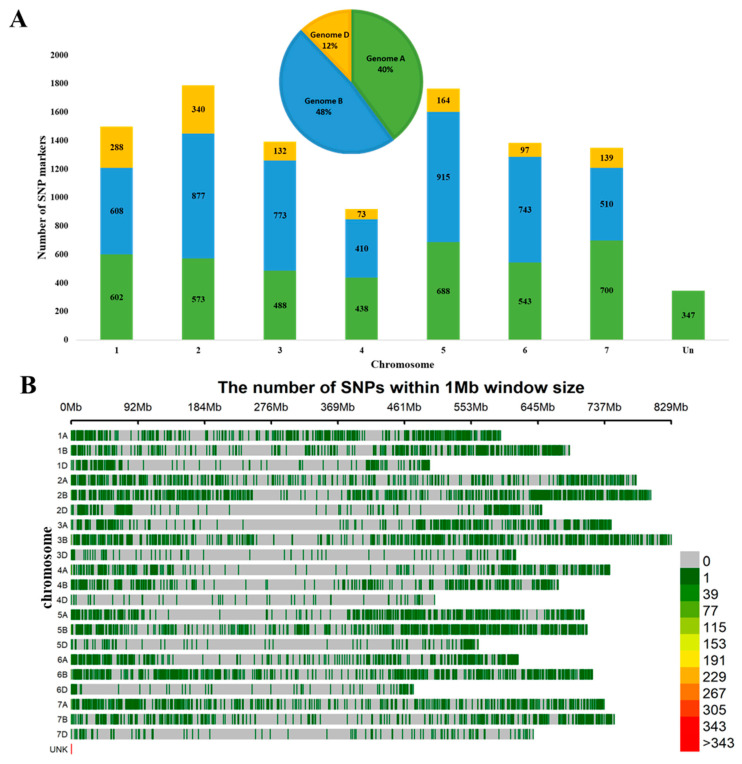
Distribution of 10,448 single-nucleotide polymorphism (SNP) markers mapped to the wheat genome: (**A**) the distribution of SNPs on seven chromosomal groups and the numbers of mapped SNPs on subgenomes A, B, and D; (**B**) the density plot of SNP markers in wheat chromosomes.

The GWAS analysis was conducted using 10,448 polymorphic SNP markers, of which 40% were mapped to the A genome, 48% to the B genome, and 12% to the D genome (Figure 3A). The minimum number of SNPs (73) was assigned to chromosome 4D, while the maximum number of SNPs was assigned to chromosome 5B (915). Homoeologous group 2 chromosomes contained the largest number of markers, at 1790 SNP markers, having a subgenome A of 573 SNPs, subgenome B of 877 SNPs, and subgenome D of 340 SNPs. Homoeologous group 4 chromosomes had the smallest number, with only 921 markers, with a subgenome A of 438 SNPs, subgenome B of 410 SNPs, and subgenome D of 73 SNPs (Figure 3A). The smallest size was found in chromosome 6D (461 Mb), and the longest was found in chromosome 3B (829 Mb). 

The results of the population structure NAM population and STRUCTURE Harvester analyses suggested that K = 3 was the optimal number of clusters for studying 290 RILs (Figure S1). The estimated r^2^ values for all pairs of linked SNP loci were used to assess the extent of LD decay in this study. As expected, the r^2^ value decreased as the physical distance between markers increased (Figure S2).

### 3.3. Identification of Marker–Trait Associations for Studied Traits

In two regions, the GWAS for four studied traits led to the detection of 74 significant quantitative trait nucleotides (QTNs) for two or more environments (Table 6 and Table S3). Specifically, 28 and 11 QTLs were identified only at the KRIAPI and SPCGF. A comparison of the GWAS results from both regions revealed that 35 QTLs were notably significant in both regions (Table 6). The majority of QTLs were localized on the chromosomes of genome B (30), followed by genomes A (29) and D (8). In the studied traits, the number of identified QTLs varied from 12 for PL to 26 for HD (Table 6). 

The GWAS detected 74 significant QTLs, 26 for HD, 22 for SMD, 14 for PH, and 12 for PH (Table 7).

**Figure 4:**
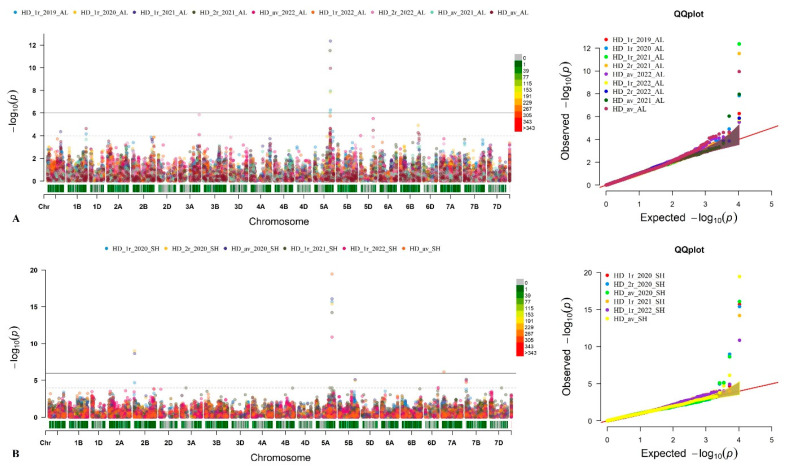
Manhattan and quantile–quantile plots (Q-Q) for the heading date (HD) in the genome-wide association study panel phenotyped in the Almaty region, KRIAPI (**A**), and Akmola region, SPCGF (**B**).

Among the identified QTLs for HD, a total of 26 were stable (QTLs found in two or more environments), with 8 and 5 of these detected at the KRIAPI and SPCGF, respectively. Thirteen of these QTLs were common to both regions. Notably, the most significant *p*-value of 3.56 × 10^−20^ was observed for chromosome 5A, detected in both regions (Table 7 and Table S3). Furthermore, *AX-94654737* exhibited detection in both regions with a PVE of 19.33%. Table 7 and visual representations are provided in Manhattan plots and Q–Q plots in Figure 4A,B for further details.

The effect of each QTL varied significantly, with the highest value observed for *AX-94681430* (−4.3 days), explained by the phenotypic variation (PVE) of 14.25% detected at the SPCGF. Another notable QTL, *AX-95122517,* was identified at the KRIAPI and had a *p*-value from 3.04 × 10^−6^, with a phenotypic variation of 30.83% (Table 7 and Table S3). 

For SMD, 22 stable QTLs were identified, with 8 and 2 detected at the KRIAPI and SPCGF, respectively. The most significant *p*-value (1.49 × 10^−11^) was observed for chromosome 7B in both regions (Table 7 and Table S3). Additionally, *AX-94510416* was detected in both regions with a PVE of 41.36%.

Regarding PL and PH, 14 and 12 QTLs were identified, respectively. The most significant QTL for PL was located on chromosome 6B and was significant in the 2022 season at the KRIAPI, with a PVE of 22.7% (Table 7 and Table S3). Furthermore, *AX-95179073* was detected in both regions and mapped to chromosome 7A with a PVE of 20.11%. All results obtained were noted to hold significance and warrant consideration in breeding projects associated with plant adaptation and yield-related traits.

## 4. Discussion

Breeding programs focused on increased yield often include optimizing additional key agronomic traits like plant height (PH) and the date of heading (HD) [69,70]. The ideal combination for HD and PH can vary significantly depending on local environmental conditions. Therefore, managing these traits poses a challenge because of their interconnected nature, where changes in one trait can affect others [71,72,73,74].

In the present study, 290 RILs of the NAM population were tested in two different contrasting parts of Kazakhstan, at the KRIAPI (southeast) and SPCGF (north). The comparative analysis of climate conditions (rainfall and temperature) showed that higher precipitation significantly contributes to increased productivity (Table 2). The assessment of the studied traits noted a large grain yield difference between the two regions (Figure 1, Table 3). The Pearson correlation analysis for average data over three years (2020–2022) showed that early HD, late SMD, taller PH, and longer PL were significantly favorable for higher YM2 at the KRIAPI but insignificant at the SPCGF. The controversial correlations among the studied traits at the SPCGF were most probably affected by the amount of rainfall at the early plant developmental stages. In favorable plant growth conditions in 2020, the YM2 negatively correlated with HD. Also, the annual assessment of correlation results at the SPCGF suggested that YM2 positively correlates with PH in two out of three years (Table 5). Therefore, early flowering time and taller plants are more favorable for higher seed productivity in both contrasting regions. 

The analysis of the average data of YM2 showed that 11 and 60 RILs showed higher values than local check cultivars at the KRIAPI and SPCGF, respectively. Two RILs, NAM-326 (Paragon × Wylakatchem-092) and NAM-138 (Paragon × Watkins349-027), showed higher yields in both regions than the local comparison check cultivars under all studied conditions (Table S1); these can be used for further wheat breeding projects in Kazakhstan. The phenotypic data presented in the current study showed that the NAM population is a valuable resource for improving agronomic traits. 

The GWAS analyses of the NAM population in the two regions led to the identification of 74 QTLs in the four traits related to plant adaptation. Notably, the largest number of QTL was identified for HD, SMD, and PH, which shows a wide range of phenotypic variations in these traits in the two regions. The largest number of QTLs were identified for HD (26 QTLs) located on chromosomes 1A (2 QTLs), 1B, 2A (4 QTLs), 2B (2 QTLs), 3A, 3B, 3D, 4A, 5A (3 QTLs), 5B, 5D, 6A, 6B, 6D, 7A, and 7B (2 QTLs). The most significant QTL (*AX-94654737*) with a *p*-value of 3.56 × 10^−20^ was observed for chromosome 5A (588,761,524 bp) detected in both regions (Table 7 and Table S3). The analysis aimed at identifying putative candidate genes using the reference genome in the Wheat Ensembl database revealed that the QTL was situated at 588,761,524 bp on chromosome 5A. Within this position, *TraesCS5A02G392700* was identified, encoding a protein annotated as an *ABC* transporter. Interestingly, in the literature, this protein has been associated with the wheat resistance gene *Lr34* [75]. This protein in the wheat ABC transporter *Lr34*, a member of the G subfamily, is known to confer partial, durable, and broad-spectrum resistance against several biotrophic fungi such as powdery mildew, leaf rust, or stem rust. Initially, it was assumed that the *Vrn-A1* gene was located at this position. Although, the physical position of *Vrn-A1* spans from 587,411,454 bp to 587,423,416 bp, with a difference of approximately 1,350,070 bp. LD analysis for chromosome 5A, which spans 6,057,956 bp, revealed that the Vrn-A1 gene is relatively close to other loci on the chromosome. Another *Vrn* gene, *Vrn-B3*, was identified within the QTL (*AX-94810990*) located at 9,702,461 bp on chromosome 7A. This position closely matches the physical position of *Vrn-B3*, which spans from 9,702,354 bp to 9,704,354 bp. *Vrn-B3* belongs to the *Vrn1* gene family, which comprises vernalization genes that regulate wheat flowering. Specifically, *Vrn-B3* promotes the transition from vegetative to reproductive growth in response to vernalization. It acts as a repressor of flowering, and its expression is downregulated by exposure to cold temperatures [76,77].

The literature survey suggested that 16 of the 74 QTLs identified in our study had also been detected in previous QTL mapping studies and GWASs for all studied traits (Table S4) [78,79,80,81,82,83,84,85,86,87,88,89,90,91]. The majority of these matches were found for PH (eight QTLs), followed by HD (five QTLs), SMD (two QTLs), and PL (one QTLs) (Table S4). Three associations associated with PH were identical to the genetic positions of QTLs identified in analyses of eight traits using 94 RILs of the mapping population of Pamyati Azieva × Paragon, tested in Kazakhstan’s northern and southern regions [78]. Three QTNs (*AX-95255993*, *AX-94504542*, * AX-94654737*) associated with HD had similar physical positions to QTLs identified in the GWAS of agronomic and quality traits in a NAM population to exploit the genetic diversity of the USDA-ARS NSGC [44]. 

The significant SNPs in the detected QTLs were analyzed to identify putative candidate genes using the annotated Chinese Spring reference genome [92] in the Wheat Ensembl database [67]. The results showed that out of the 74 identified QTLs, 51 were located in genic positions (Table S3). An analysis of these 51 genes suggested that most were associated with controlling plant growth, development, and abiotic/biotic stress tolerance [93,94,95,96,97,98,99,100]. For example, two QTLs were associated with HD (*AX-94943644*) and SMD (*AX-94483125*), where significant SNPs were aligned with F-box-domain-containing proteins. The F-box proteins regulate plant development and control flowering time [93,94]. It was determined that *AX-94634646*, associated with PL, encodes *Domain of unknown function* (DUF)-domain-containing proteins, which play a role in plant development and fitness in rice [95]. The list of genes and proteins related to stress resistance/tolerance includes protein kinase superfamily protein (*TraesCS1B02G469400*, *TraesCS2B02G001600*, *TraesCS2D02G474300*, *TraesCS3B02G424200*) [96], a zinc finger protein (*TraesCS7A02G173200*) [97], and a CSC1-like protein RXW8 (*TraesCS5A02G012500*) associated with chilling tolerance [98]. In addition, a list of genes related to stress drought tolerance included *TraesCS2B02G608300* (Potassium efflux antiporter), *TraesCS3A02G493000* (EF-hand-domain-containing protein) [99], and *TraesCS1D02G072900* (*WRKY26* transcription factor) [100]. 

The alignment of associations identified in this study with previously published reports confirms the results’ reliability. While the identified QTLs should undergo further validation in subsequent experiments, there is a promising indication that most presumably novel associations hold significance for plant adaptation-related traits. Consequently, the SNPs identified within the detected QTLs will likely have significant value for successful application in marker-assisted wheat breeding.

## 5. Conclusions

The analysis of 290 RILs of the NAM population in two contrasting regions of Kazakhstan (north and southeast) indicated that early heading time and taller plants are more favorable for grain productivity. The assessment of the average YM2 values suggested that 11 and 60 RILs showed higher values than local check cultivars in the southeast and north regions, respectively. Hence, the phenotypic data showed that the NAM population is valuable for improving agronomic traits. The GWAS of the NAM population in the two regions allowed the identification of 74 QTLs in the four traits related to plant adaptation (HD, SMD, PH, and PL). The largest number of QTLs were identified for HD (26 QTLs), including two QTLs in the vicinity of the physical positions of *Vrn-A1* (chromosome 5A) and *Vrn-B3* (chromosome 7A). The study provided a valuable data source for the search for new genes associated with wheat plant adaption. 

## Figures and Tables

**Table 1:** The list of accessions used as parental lines for the developed nested association mapping population.

Cultivars	Origin (Countries)	Mapping Population	Number of RIL
Watkins34	India (Asia)	Paragon × Watkins34	8
Watkins141	China (Asia)	Paragon × Watkins141	10
Watkins216	Morocco (North Africa)	Paragon × Watkins216	10
Watkins223	Burma (Asia)	Paragon × Watkins223	11
Watkins254	Morocco (North Africa)	Paragon × Watkins254	13
Watkins264	Canary Islands (Western Europe)	Paragon × Watkins264	13
Watkins273	Spain (Western Europe)	Paragon × Watkins273	14
Watkins291	Cyprus (Western Europe)	Paragon × Watkins291	14
Watkins292	Cyprus (Western Europe)	Paragon × Watkins292	11
Watkins299	Türkiye (Middle East)	Paragon × Watkins299	11
Watkins349	Bulgaria (Eastern Europe)	Paragon × Watkins349	12
Watkins396	Portugal (Western Europe)	Paragon × Watkins396	10
Watkins397	Portugal (Western Europe)	Paragon × Watkins397	13
Watkins398	Palestine (Middle East)	Paragon × Watkins398	9
Watkins420	India (Asia)	Paragon × Watkins420	12
Watkins546	Spain (Western Europe)	Paragon × Watkins546	13
Watkins566	Greece (Western Europe)	Paragon × Watkins566	12
Watkins685	Spain (Western Europe)	Paragon × Watkins685	12
Watkins811	Tunisia (North Africa)	Paragon × Watkins811	13
BAJ		Paragon × BAJ	15
CIMCOG 47	Mexico	Paragon × CIMCOG 47	16
CIMCOG 49	Mexico	Paragon × CIMCOG 49	16
Wylakatchem	Australia	Paragon × Wylakatchem	15
PFAU	Mexico	Paragon × PFAU	7

**Table 2:** Location, environment, and weather data for the two study regions in Kazakhstan.

Site/Region	KRIAPI (Almaty Region, Southeast Kazakhstan)				SPCGF (Akmola Region, Northern Kazakhstan)		
Latitude/Longitude	43°21′/76°53′				51°40′/71°00′		
Soil type	Light chestnut (humus 2.0–2.5%)				Southern carbonate chernozem (humus 3.6%)		
Conditions	Rainfed				Rainfed		
Year	2019	2020	2021	2022	2020	2021	2022
Annual rainfall, mm	299	279	183	250	426	112	117
Mean temperature, °C	19.8	19.8	21.8	22.2	19.2	18.0	18.4
Max temperature, °C	27.0	24.2	27.4	26.5	20.7	20.4	21.1
Min temperature, °C	12.9	14.2	12.4	16.7	17.6	14.7	15.7

Note: KRIAPI—Kazakh Research Institute of Agriculture and Plant Industry; SPCGF—Alexandr Barayev Scientific-Production Center for Grain Farming.

**Table 3:** Phenotypic distribution of trait nested association mapping (NAM) of 24 families in two regions.

NAM Population/Region	HD, days		SMD, days		PH, cm		PL, cm		YM2_g/m^2^	
	KRIAPI	SPCGF	KRIAPI	SPCGF	KRIAPI	SPCGF	KRIAPI	SPCGF	KRIAPI	SPCGF
Paragon × Watkins34	53.4 ± 0.65	37.7 ± 1.00	34.5 ± 0.58	43.9 ± 0.54	76.3 ± 1.20	42.3 ± 0.99	30.8 ± 1.23	18.1 ± 0.50	421.5 ± 30.49	188.0 ± 16.28
Paragon × Watkins141	62.7± 0.76	47.2 ± 1.33	31.6 ± 0.46	42.5 ± 0.78	86.2 ± 1.26	54.0 ± 1.96	30.4 ± 0.80	21.0 ± 0.99	299.9 ± 17.67	271.7 ± 17.70
Paragon × Watkins216	59.2 ± 0.48	43.7 ± 1.18	32.6 ± 0.26	42.5 ± 0.62	86.2 ± 1.21	52.9 ± 1.25	33.0 ± 0.91	22.1 ± 0.71	262.0 ± 12.93	206.3 ± 9.66
Paragon × Watkins223	55.1 ± 0.77	37.7 ± 0.80	34.4 ± 0.39	43.8 ± 0.61	83.1 ± 1.20	45.6 ± 0.93	32.4 ± 0.84	19.6 ± 0.73	449.4 ± 33.14	223.9 ± 12.56
Paragon × Watkins254	61.5 ± 0.83	44.7 ± 1.13	31.6 ± 0.53	43.0 ± 0.30	81.1 ± 2.26	53.1 ± 1.80	30.2 ± 1.46	22.3 ± 0.99	332.8 ± 26.15	234.0 ± 11.45
Paragon × Watkins264	57.4 ± 0.53	42.1 ± 0.86	33.4 ± 0.35	43.3 ± 0.23	87.7 ± 1.84	52.6 ± 1.00	36.8 ± 0.89	23.2 ± 0.73	389.7 ± 20.47	264.6 ± 23.04
Paragon × Watkins273	57.0± 0.67	42.9± 1.20	33.4 ± 0.24	44.4 ± 0.47	86.7 ± 1.80	51.1 ± 1.34	34.4 ± 0.91	22.1 ± 0.60	305.4 ± 28.33	228.6 ± 26.03
Paragon × Watkins291	59.0 ± 0.82	42.0 ± 0.81	32.1 ± 0.42	42.9 ± 0.18	84.5 ± 1.96	50.6 ± 1.42	29.8 ± 0.74	20.7 ± 0.74	392.1 ± 22.60	237.9 ± 13.54
Paragon × Watkins292	59.0 ± 0.83	45.2 ± 1.42	31.6 ± 0.36	42.0 ± 0.75	81.4 ± 1.20	49.2 ± 1.73	31.0 ± 1.24	19.8 ± 1.09	375.1 ± 15.81	177.2 ± 17.76
Paragon × Watkins299	57.8 ± 0.56	39.6 ± 0.83	32.6 ± 0.44	44.2 ± 0.24	82.1 ± 1.43	46.0 ± 1.26	33.3 ± 0.76	20.2 ± 0.63	423.6 ± 34.68	240.3 ± 12.00
Paragon × Watkins349	61.0 ± 0.51	42.0 ± 0.59	32.5 ± 0.32	43.4 ± 0.15	87.0 ± 1.85	53.5 ± 1.27	32.7 ± 0.48	24.0 ± 0.61	439.1 ± 27.96	288.8 ± 20.49
Paragon × Watkins396	63.2 ± 1.01	44.7 ± 0.96	31.4 ± 0.56	43.3 ± 0.27	79.2 ± 1.98	55.1 ± 1.54	31.3 ± 1.17	24.2 ± 0.88	331.6 ± 26.65	310.2 ± 19.02
Paragon × Watkins397	59.0 ± 0.83	41.8 ± 0.83	32.8 ± 0.52	43.7 ± 0.21	90.1 ± 1.55	52.7 ± 1.29	35.1 ± 0.99	23.5 ± 0.42	387.8 ± 23.74	247.4 ± 17.50
Paragon × Watkins398	58.2 ± 0.47	42.7 ± 1.04	33.3 ± 0.27	42.5 ± 0.49	79.7 ± 1.75	49.5 ± 1.67	31.4 ± 1.60	21.1 ± 0.71	391.3 ± 24.11	241.8 ± 16.33
Paragon × Watkins420	59.5 ± 0.73	43.9 ± 1.94	32.9 ± 0.53	43.4 ± 0.61	80.8 ± 1.30	52.0 ± 1.49	30.8 ± 1.14	22.5 ± 0.77	392.9 ± 28.24	289.8 ± 14.00
Paragon × Watkins546	59.7 ± 0.87	42.3 ± 1.00	34.2 ± 0.46	42.5 ± 0.56	87.7 ± 1.75	55.4 ± 0.78	33.5 ± 0.96	24.6 ± 0.71	433.2 ± 20.52	293.9 ± 12.24
Paragon × Watkins566	60.4 ± 0.49	45.5 ± 1.01	32.9 ± 0.32	42.7 ± 0.54	89.2 ± 2.09	53.9 ± 0.69	31.5 ± 0.73	22.4 ± 0.67	359.5 ± 13.15	252.5 ± 15.94
Paragon × Watkins685	57.8 ± 0.52	43.5 ± 1.23	31.9 ± 0.38	41.8 ± 0.74	80.8 ± 1.50	50.2 ± 0.86	31.8 ± 0.44	22.3 ± 0.60	347.0 ± 9.45	202.3 ± 21.86
Paragon × Watkins811	56.4 ± 0.34	43.6 ± 0.88	32.0 ± 0.18	43.0 ± 0.21	85.6 ± 2.27	49.7 ± 1.28	33.6 ± 1.38	21.5 ± 0.79	407.5 ± 19.53	245.4 ± 13.51
Paragon × BAJ	56.0 ± 0.59	41.4 ± 0.71	32.2 ± 0.25	43.0 ± 0.20	60.5 ± 1.37	41.1 ± 1.00	21.7 ± 0.43	14.5 ± 0.50	361.5 ± 18.76	290.1 ± 21.00
Paragon × CIMCOG 47	54.7 ± 0.34	40.1 ± 0.47	32.7 ± 0.23	42.9 ± 0.29	61.6 ± 1.53	40.9 ± 1.05	25.0 ± 0.77	17.0 ± 0.56	403.3 ± 19.31	297.6 ± 14.18
Paragon × CIMCOG 49	55.9 ± 0.55	40.4 ± 0.53	32.9 ± 0.37	44.0 ± 0.21	65.4 ± 1.66	45.7 ± 1.15	26.1 ± 0.83	18.9 ± 0.74	376.8 ± 24.98	237.7 ± 19.08
Paragon × PFAU	52.8 ± 0.69	38.1 ± 0.82	33.5 ± 0.49	44.3 ± 0.42	59.3 ± 2.61	38.3 ± 2.01	21.8 ± 1.48	15.2 ± 1.32	208.5 ± 20.90	269.9 ± 28.27
Paragon × Wylakatchem	56.5 ± 0.45	40.0 ± 0.39	32.4 ± 0.26	43.2 ± 0.18	64.1 ± 1.91	41.8 ± 0.84	25.4 ± 0.96	17.1 ± 0.68	328.5 ± 23.61	298.7 ± 13.23

Note: HD, days—heading date; SMD, days—seed maturation date; PH, cm—plant height; PL, days—peduncle length; YM2, g/m^2^—yield per m^2^; KRIAPI—Kazakh Research Institute of Agriculture and Plant Industry; SPCGF—Alexandr Barayev Scientific-Production Center for Grain Farming.

**Table 4:** Analysis of variance (ANOVA) results for studied traits of nested association mapping population grown in Kazakhstan.

Traits	Factor	Df	Sum Sq	Mean Sq	F-Value	h_b_^2^
HD, days	Genotype (G)	279	30,899	111	14.59 ***	29.8%
	Environment (E)	2	47,631	23,815	3138.26 ***	
	G:E	558	18,932	34	4.47 ***	
	Residuals	840	6375	8		
SMD, days	Genotype (G)	279	3380	12	1.97 ***	2.2%
	Environment (E)	2	136,499	68,250	11,093.20 ***	
	G:E	558	5366	10	1.56 ***	
	Residuals	840	5168	6		
PH, cm	Genotype (G)	279	114,177	409	11.96 ***	13.9%
	Environment (E)	2	624,924	312,462	9128.80 ***	
	G:E	558	52,838	95	2.77 ***	
	Residuals	840	28,752	34		
PL, cm	Genotype (G)	279	27,909	100	5.09 ***	10.0%
	Environment (E)	2	213,112	106,556	5421.09 ***	
	G:E	558	20,949	38	1.91 ***	
	Residuals	840	16,511	20		
YM2, g/m^2^	Genotype (G)	279	8,638,103	30,961	5.79 ***	14.9%
	Environment (E)	2	30,095,833	15,047,916	2815.83 ***	
	G:E	558	14,653,053	26,260	4.91 ***	
	Residuals	840	4,488,995	5344		

Note: *p*-values are provided with significance level indicated by the asterisks; *** *p* < 0.001; HD, days—heading date; SMD, days—seed maturation date; PH, cm—plant height; PL, days—peduncle length; YM2, g/m^2^—yield per m^2^.

**Table 5:** Pearson’s correlation index by years (2020–2022) of five studied traits in spring wheat nested association mapping population grown in the southeast and north of Kazakhstan.

KRIAPI					SPCGF				
2020									
	SMD	PH	PL	YM2		SMD	PH	PL	YM2
HD	−0.44 ***	0.15 *	−0.12 *	0.02 ns	HD	−0.06 ns	0.39 ***	0.16 **	−0.30 ***
SMD		0.31 ***	0.28 ***	0.21 ***	SMD		0.02 ns	0.01 ns	−0.03 ns
PH			0.75 ***	0.32 ***	PH			0.68 ***	−0.09 ns
PL				0.22 ***	PL				0.01 ns
2021									
	SMD	PH	PL	YM2		SMD	PH	PL	YM2
HD	−0.70 ***	0.12 *	0.02 ns	−0.46 ***	HD	−0.59 ***	0.48 ***	0.21 ***	0.05 ns
SMD		0.03 ns	0.11 ns	0.23 ***	SMD		−0.22 ***	−0.05 ns	−0.10 ns
PH			0.80 ***	0.30 ***	PH			0.76 ***	0.29 ***
PL				0.26 ***	PL				0.24 ***
2022									
	SMD	PH	PL	YM2		SMD	PH	PL	YM2
HD	−0.60 ***	0.35 ***	−0.02 ns	−0.31 ***	HD	−0.79 ***	0.22 ***	0.13 *	0.13 *
SMD		−0.21 ***	−0.01 ns	0.24 ***	SMD		−0.19 **	−0.13 *	−0.13 *
PH			0.60 ***	0.02 ns	PH			0.72 ***	0.23 ***
PL				0.27 ***	PL				0.12 *
mean									
	SMD	PH	PL	YM2		SMD	PH	PL	YM2
HD	−0.59 ***	0.33 ***	−0.06 ns	−0.09 ns	HD	−0.79 ***	0.22 ***	0.13 ***	0.13 ns
SMD		0.10 ns	0.32 ***	0.23 ***	SMD		−0.19 *	−0.13 ns	−0.13 ns
PH			0.76 ***	0.25 ***	PH			0.72 ***	0.23 ns
PL				0.27 ***	PL				0.12 ns

Note: *p*-values are provided with significance level indicated by the asterisks; * *p* < 0.05, ** *p* < 0.01, *** *p* < 0.001; ns—not significant; HD—heading date (days); SMD—seed maturation date (days); PH—plant height (cm); PL—peduncle length (cm); YM2—yield per m^2^ (g/m^2^). KRIAPI—Kazakh Research Institute of Agriculture and Plant Industry; SPCGF—Alexandr Barayev Scientific-Production Center for Grain Farming.

**Table 6:** Summary of identified marker–trait associations in the NAM population spring wheat based on field performance in the two locations.

Trait	Identified QTL	KRIAPI	SPCGF	Both Regions
Heading date (HD, days)	26	8	5	13
Seed maturation date (SMD, days)	22	8	2	12
Plant height (PH, cm)	14	7	2	5
Peduncle length (PL, cm)	12	5	2	5
Total	74	28	11	35

Note: KRIAPI—Kazakh Research Institute of Agriculture and Plant Industry; SPCGF—Alexandr Barayev Scientific-Production Center for Grain Farming.

**Table 7:** The list of QTLs for four studied traits identified using 290 RILs of the NAM population in the two regions.

QTLs	SNP	Chr.	Pos., bp	*p*-Value	Regions
*QHD.ta.NAM.ipbb-1A.1*	*AX-94561041*	1A	41,901,010	6.36 × 10^−4^	both
*QHD.ta.NAM.ipbb-1A.2*	*AX-94768074*	1A	474,699,818	4.45 × 10^−5^	KPIAPI
*QHD.ta.NAM.ipbb-1B*	*AX-94592638*	1B	678,266,710	2.33 × 10^−5^	KPIAPI
*QHD.ta.NAM.ipbb-2A.1*	*AX-95255993*	2A	31,811,157	5.39 × 10^−4^	SPCGF
*QHD.ta.NAM.ipbb-2A.2*	*AX-95098442*	2A	43,299,265	3.05 × 10^−4^	both
*QHD.ta.NAM.ipbb-2A.3*	*AX-94665800*	2A	603,549,569	2.54 × 10^−4^	both
*QHD.ta.NAM.ipbb-2A.4*	*AX-94504542*	2A	729,298,590	4.50 × 10^−4^	KPIAPI
*QHD.ta.NAM.ipbb-2B.1*	*AX-94681430*	2B	18,941,804	1.05 × 10^−9^	SPCGF
*QHD.ta.NAM.ipbb-2B.2*	*AX-94393895*	2B	788,664,980	1.32 × 10^−4^	both
*QHD.ta.NAM.ipbb-3A*	*AX-94701190*	3A	719,763,842	1.38 × 10^−6^	both
*QHD.ta.NAM.ipbb-3B*	*AX-95249280*	3B	571,763,709	7.37 × 10^−4^	KPIAPI
*QHD.ta.NAM.ipbb-3D*	*AX-94713011*	3D	484,808,321	6.69 × 10^−4^	both
*QHD.ta.NAM.ipbb-4A*	*AX-95633345*	4A	707,039,327	1.82 × 10^−4^	KPIAPI
*QHD.ta.NAM.ipbb-5A.1*	*AX-94603117*	5A	476,763,775	1.19 × 10^−4^	both
*QHD.ta.NAM.ipbb-5A.2*	*AX-94654737*	5A	588,761,524	3.56 × 10^−20^	both
*QHD.ta.NAM.ipbb-5A.3*	*AX-94725943*	5A	673,709,691	4.11 × 10^−5^	both
*QHD.ta.NAM.ipbb-5B.1*	*AX-95256298*	5B	460,267,677	3.01 × 10^−4^	SPCGF
*QHD.ta.NAM.ipbb-5B.2*	*AX-94386712*	5B	591,836,342	7.67 × 10^−6^	both
*QHD.ta.NAM.ipbb-5D*	*AX-95122517*	5D	462,988,586	3.04 × 10^−6^	KPIAPI
*QHD.ta.NAM.ipbb-6A*	*AX-94943644*	6A	140,607,311	9.31 × 10^−4^	SPCGF
*QHD.ta.NAM.ipbb-6B*	*AX-94570953*	6B	658,818,818	1.23 × 10^−5^	both
*QHD.ta.NAM.ipbb-6D*	*AX-94562028*	6D	468,842,171	9.40 × 10^−5^	SPCGF
*QHD.ta.NAM.ipbb-7A*	*AX-94755544*	7A	127,676,409	1.90 × 10^−4^	KPIAPI
*QHD.ta.NAM.ipbb-7B.1*	*AX-94810990*	7B	9,702,461	7.28 × 10^−6^	both
*QHD.ta.NAM.ipbb-7B.2*	*AX-94684729*	7B	676,144,642	2.37 × 10^−4^	both
*QHD.ta.NAM.ipbb-UNK*	*AX-95256830*	UNK	30,120	4.45 × 10^−4^	KPIAPI
*QSMD.ta.NAM.ipbb-1A.1*	*AX-94500759*	1A	128,626,137	1.00 × 10^−6^	KPIAPI
*QSMD.ta.NAM.ipbb-1A.2*	*AX-94964616*	1A	517,415,353	2.36 × 10^−7^	both
*QSMD.ta.NAM.ipbb-1B.1*	*AX-95208428*	1B	478,053,661	1.07 × 10^−4^	both
*QSMD.ta.NAM.ipbb-1B.2*	*AX-94610095*	1B	587,823,781	1.17 × 10^−4^	both
*QSMD.ta.NAM.ipbb-1D*	*AX-94636030*	1D	53,381,669	2.10 × 10^−4^	both
*QSMD.ta.NAM.ipbb-2A*	*AX-95099971*	2A	94,003,182	1.07 × 10^−7^	KPIAPI
*QSMD.ta.NAM.ipbb-3A.1*	*AX-94605747*	3A	54,939,425	4.85 × 10^−4^	SPCGF
*QSMD.ta.NAM.ipbb-3A.2*	*AX-94866541*	3A	568,383,306	1.65 × 10^−5^	both
*QSMD.ta.NAM.ipbb-3B.1*	*AX-94808751*	3B	431,589,634	5.62 × 10^−5^	SPCGF
*QSMD.ta.NAM.ipbb-3B.2*	*AX-94483125*	3B	781,461,038	4.12 × 10^−5^	both
*QSMD.ta.NAM.ipbb-4A*	*AX-94542577*	4A	614,111,171	1.93 × 10^−4^	both
*QSMD.ta.NAM.ipbb-5A.1*	*AX-95235821*	5A	8,237,880	5.80 × 10^−7^	KPIAPI
*QSMD.ta.NAM.ipbb-5A.2*	*AX-94690257*	5A	706,429,847	1.44 × 10^−6^	KPIAPI
*QSMD.ta.NAM.ipbb-5B.1*	*AX-94817648*	5B	25,666,462	8.85 × 10^−5^	KPIAPI
*QSMD.ta.NAM.ipbb-5B.2*	*AX-94890794*	5B	566,685,969	3.23 × 10^−5^	both
*QSMD.ta.NAM.ipbb-6B*	*AX-94609735*	6B	−1	3.04 × 10^−4^	both
*QSMD.ta.NAM.ipbb-7B*	*AX-94510416*	7B	707,698,825	1.49 × 10^−11^	both
*QSMD.ta.NAM.ipbb-7D.1*	*AX-94696494*	7D	−1	1.37 × 10^−5^	both
*QSMD.ta.NAM.ipbb-7D.2*	*AX-94747939*	7D	58,869,306	5.28 × 10^−5^	KPIAPI
*QSMD.ta.NAM.ipbb-UNK.1*	*AX-94597695*	UNK	9,920	3.04 × 10^−4^	KPIAPI
*QSMD.ta.NAM.ipbb-UNK.2*	*AX-94779279*	UNK	19,750	4.70 × 10^−8^	KPIAPI
*QSMD.ta.NAM.ipbb-UNK.3*	*AX-95254671*	UNK	30,050	3.59 × 10^−13^	both
*QPH.ta.NAM.ipbb-1A*	*AX-95104178*	1A	340,249,943	6.52 × 10^−5^	KPIAPI
*QPH.ta.NAM.ipbb-2B.1*	*AX-94818538*	2B	−1	3.14 × 10^−4^	SPCGF
*QPH.ta.NAM.ipbb-2B.2*	*AX-95150897*	2B	115,839,405	3.19 × 10^−4^	SPCGF
*QPH.ta.NAM.ipbb-2D*	*AX-94705599*	2D	577,454,929	3.20 × 10^−5^	KPIAPI
*QPH.ta.NAM.ipbb-3A*	*AX-95083017*	3A	699,419,434	4.80 × 10^−4^	KPIAPI
*QPH.ta.NAM.ipbb-3B*	*AX-95208494*	3B	661,827,596	4.15 × 10^−4^	both
*QPH.ta.NAM.ipbb-4B*	*AX-95630372*	4B	169,935,701	3.50 × 10^−4^	both
*QPH.ta.NAM.ipbb-5B.1*	*AX-94541915*	5B	−1	5.91 × 10^−5^	KPIAPI
*QPH.ta.NAM.ipbb-5B.2*	*AX-94392836*	5B	679,687,601	3.16 × 10^−5^	both
*QPH.ta.NAM.ipbb-6A.1*	*AX-94783460*	6A	127,189,675	1.38 × 10^−4^	both
*QPH.ta.NAM.ipbb-6A.2*	*AX-94575241*	6A	573,496,900	1.36 × 10^−4^	KPIAPI
*QPH.ta.NAM.ipbb-7A*	*AX-94492491*	7A	581,848,865	1.84 × 10^−5^	both
*QPH.ta.NAM.ipbb-7B*	*AX-94439304*	7B	334,455,703	2.15 × 10^−4^	KPIAPI
*QPH.ta.NAM.ipbb-UNK*	*AX-94659909*	UNK	31,410	1.18 × 10^−4^	KPIAPI
*QPL.ta.NAM.ipbb-1B*	*AX-95022601*	1B	106,765,751	1.30 × 10^−4^	KPIAPI
*QPL.ta.NAM.ipbb-2D*	*AX-94444526*	2D	30,405,035	9.41 × 10^−5^	KPIAPI
*QPL.ta.NAM.ipbb-4A*	*AX-94945797*	4A	541,340,650	1.27 × 10^−4^	both
*QPL.ta.NAM.ipbb-4B.1*	*AX-95129444*	4B	480,923,965	2.04 × 10^−4^	SPCGF
*QPL.ta.NAM.ipbb-4B.2*	*AX-95630385*	4B	609,515,886	5.45 × 10^−4^	both
*QPL.ta.NAM.ipbb-6B*	*AX-94793082*	6B	117,516,187	1.81 × 10^−6^	KPIAPI
*QPL.ta.NAM.ipbb-7A.1*	*AX-94634646*	7A	23,238,304	5.31 × 10^−4^	KPIAPI
*QPL.ta.NAM.ipbb-7A.2*	*AX-95179073*	7A	647,297,932	2.23 × 10^−6^	both
*QPL.ta.NAM.ipbb-7B.1*	*AX-94503821*	7B	−1	4.60 × 10^−4^	KPIAPI
*QPL.ta.NAM.ipbb-7B.2*	*AX-94587603*	7B	61,077,481	2.04 × 10^−5^	SPCGF
*QPL.ta.NAM.ipbb-7B.3*	*AX-94545252*	7B	133,792,764	4.94 × 10^−5^	both
*QPL.ta.NAM.ipbb-7B.4*	*AX-94505633*	7B	401,550,322	5.32 × 10^−4^	both

Note: Chr—chromosome; Pos., bp—physical position of markers; UNK—unknown chromosome; −1—unknown positions; KRIAPI—Kazakh Research Institute of Agriculture and Plant Industry; SPCGF—Alexandr Barayev Scientific-Production Center for Grain Farming.

## Data Availability

The original contributions presented in the study are included in the article/supplementary material, further inquiries can be directed to the corresponding author/s.

## Supplementary Materials

The following supporting information can be downloaded at: https://www.mdpi.com/article/10.3390/plants13182623/s1, Table S1: The raw field data at the Kazakh Research Institute of Agriculture and Plant Industry (KRIAPI, Almaty region, Southeast Kazakhstan) and Alexandr Barayev Scientific-Production Center for Grain Farming (SPCGF, Shortandy, Akmola region, Northern Kazakhstan); Table S2: Results of t-test for five studied traits between two regions in southeast and north of Kazakhstan; Table S3: The list of QTLs and genes for five studied traits identified using 290 RILs of the NAM population in the conditions of the Kazakh Research Institute of Agriculture and Plant Industry (KRIAPI, Almaty region, Southeast Kazakhstan, 2019–2022) and Alexandr Barayev Scientific-Production Center for Grain Farming (SPCGF, Shortandy, Akmola region, Northern Kazakhstan, 2020–2022); Table S4: List of identified QTLs based on the GWAS analysis of wheat collection compared to the associations revealed in previously published reports; Figure S1: Population structure of the NAM population based on 10,448 SNP markers: (A) STRUCTURE Harvester output for delta K; (B) separation of samples into clusters based on the STRUCTURE package at K = 4. The colors in the boxes represent the clusters identified in Figure S1B; Figure S2: Chromosome-wide linkage disequilibrium (LD) decay estimated for 10,448 SNPs of 290RILs of the NAM population.
