# Supplementary material for: Identification of Quantitative Trait Loci Associated with Plant Adaptation Traits Using Nested Association Mapping Population
Source: Plants (Basel). 2024 Sep 20;13(18):2623. doi: 10.3390/plants13182623 (PMC11435412; doi:10.3390/plants13182623)
Supplement: Supplementary file 1 [file plants-13-02623-s001.zip › Table S2.pdf]

Table S2. Results of t-test for five studied traits between two regions in the southeast and north of Kazakhstan

Year/Traits	HD, days	SMD, days	PH, cm	PL, cm	YM2, g/m^2
2020	5,91741E-61	5,8112E-239	2,943E-126	2,17E-29	2,28E-31
2021	2,6199E-124	3,7077E-267	0,585740174	1,88E-37	1,6E-138
2022	0	9,8408E-131	1,5158E-243	4,2E-230	1,2E-137
average	2,131E-220	0	5,7956E-164	5,3E-102	1,43E-53

Note: HD, days – heading date, SMD, days – seed maturation date, PH, cm – plant height, PL,cm – peduncle length, YM2, g/m^2 – yield per m^2.
