# Supplementary figures and images for: Identification of Quantitative Trait Loci Associated with Plant Adaptation Traits Using Nested Association Mapping Population
Source: Plants (Basel). 2024 Sep 20;13(18):2623. doi: 10.3390/plants13182623 (PMC11435412; doi:10.3390/plants13182623)

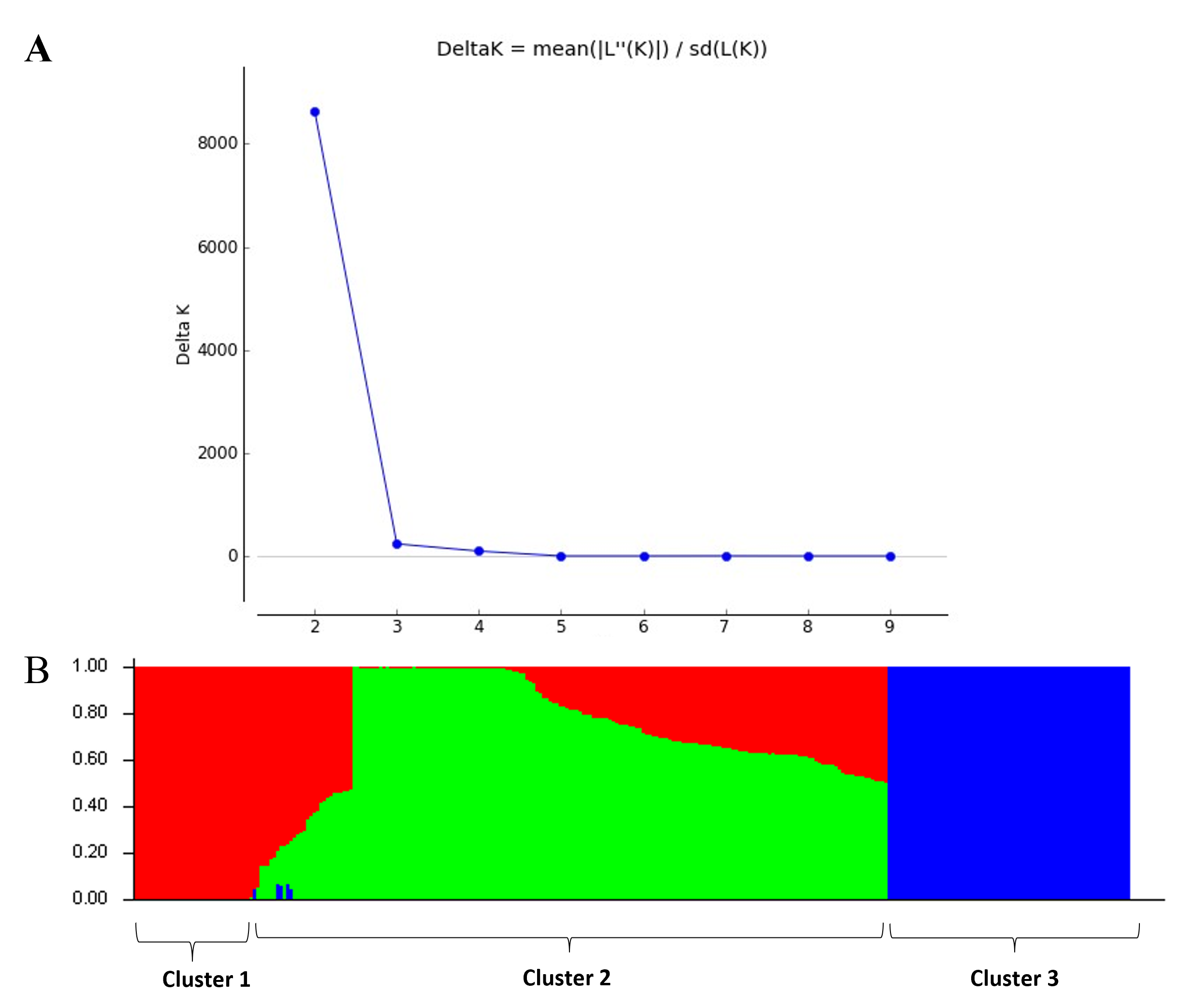

Supplement: Supplementary file 1 [file plants-13-02623-s001.zip › Figure S1.png]

**1A**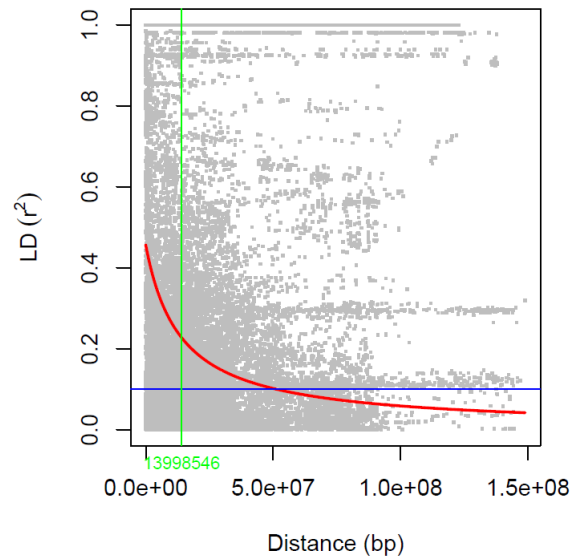**1B**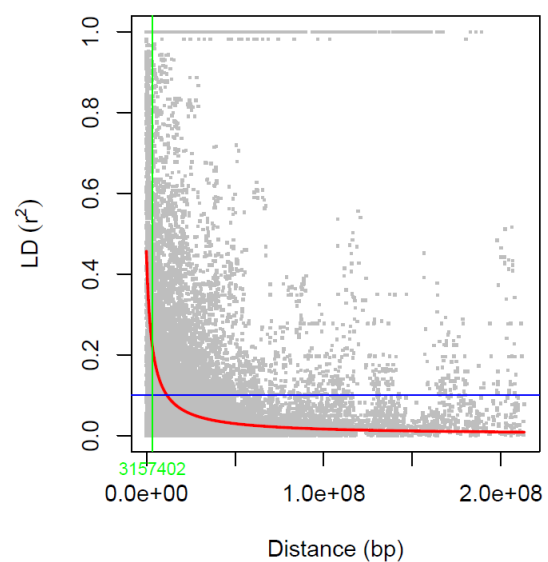**1D**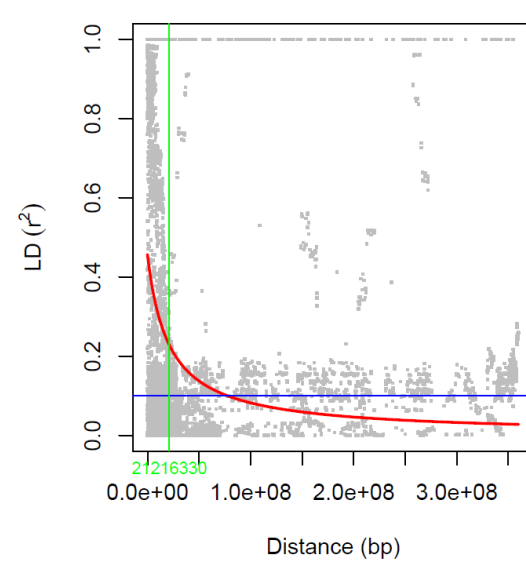**2A**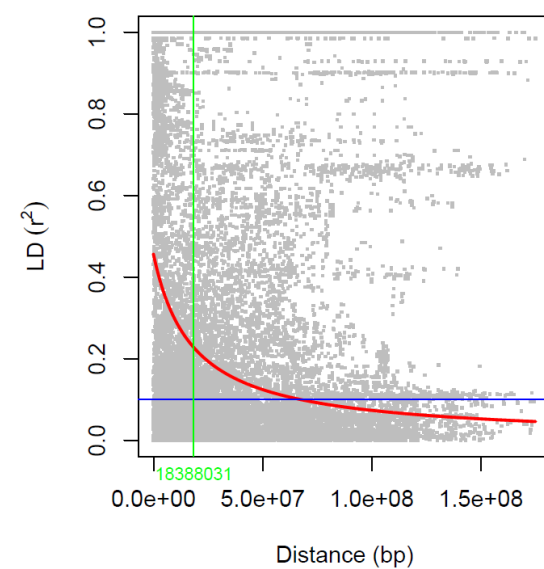**2B**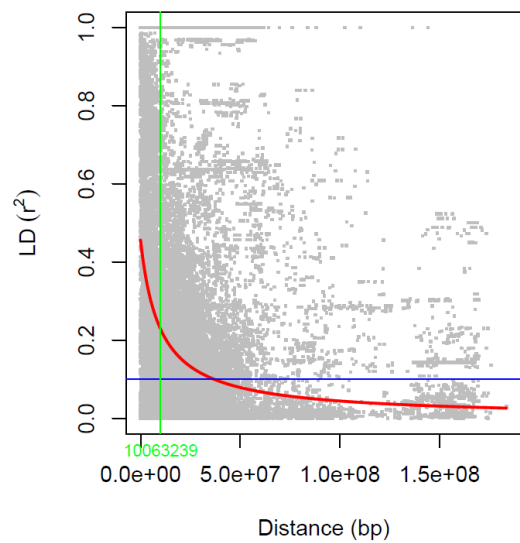**2D**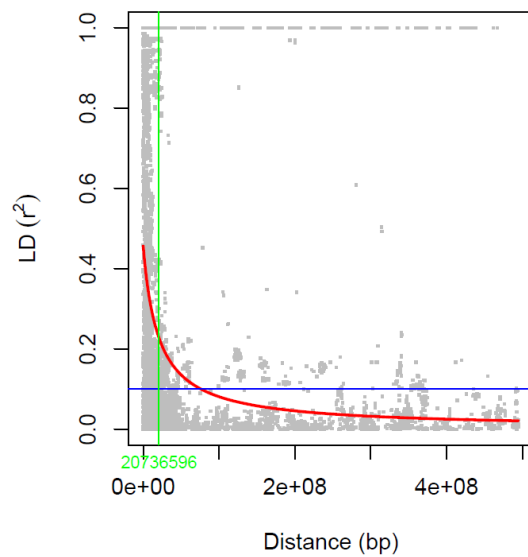**3A**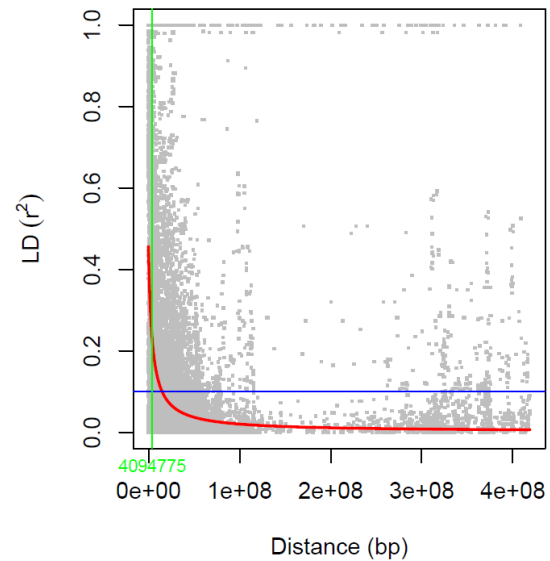**3B**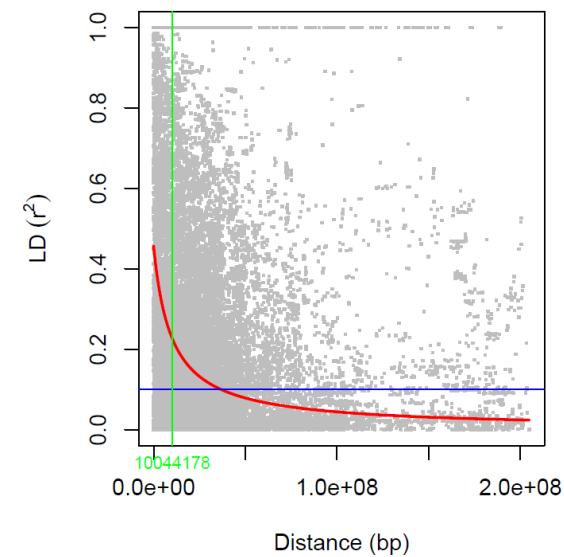

**3D**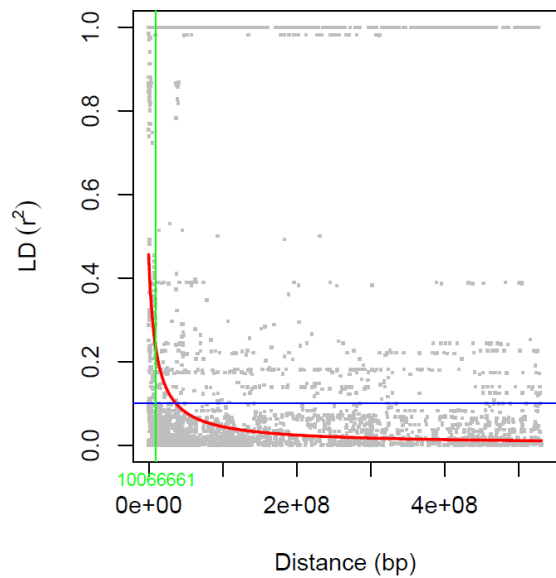**4A**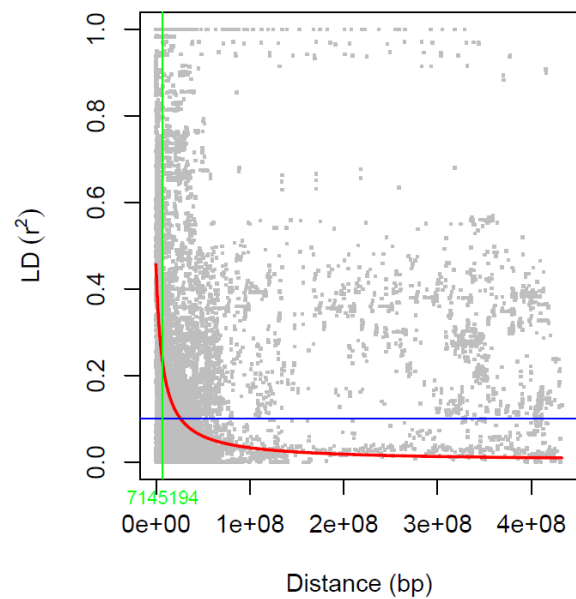**4B**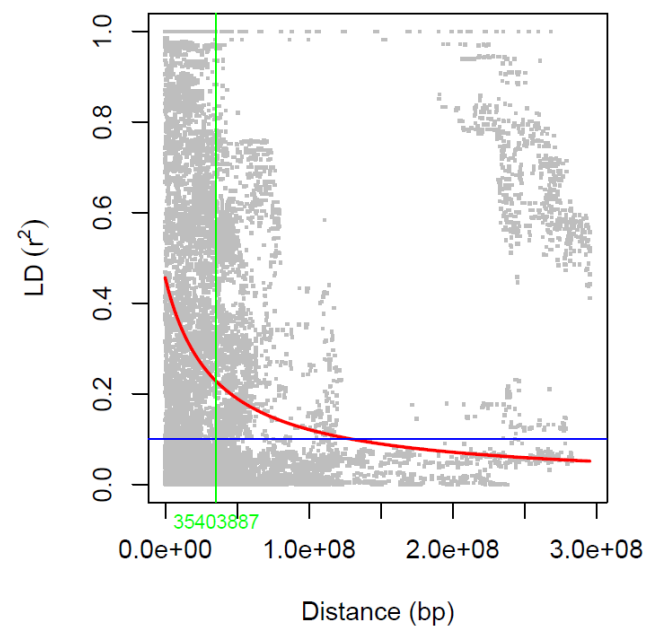**4D**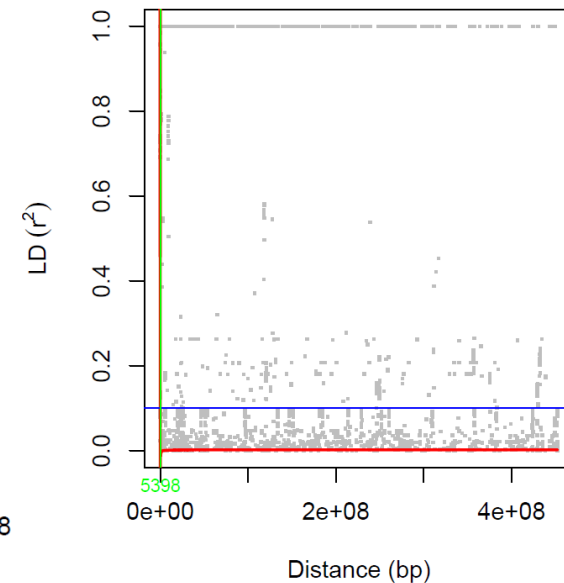**5A**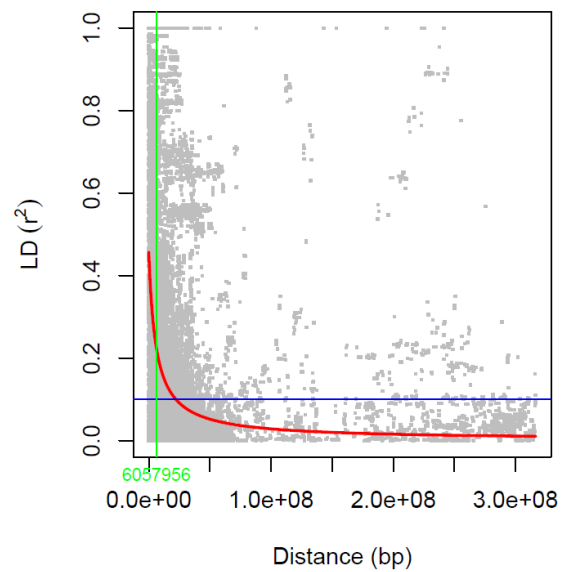**5B**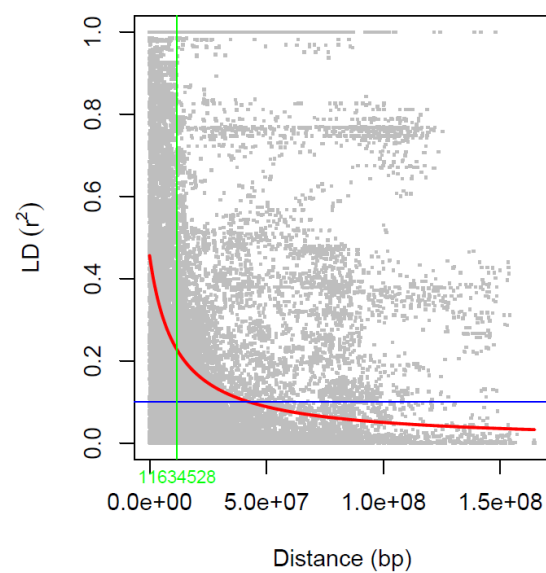**5D**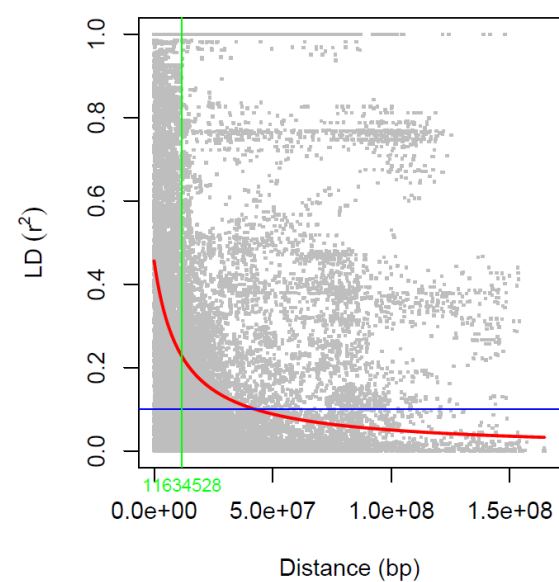

**6A**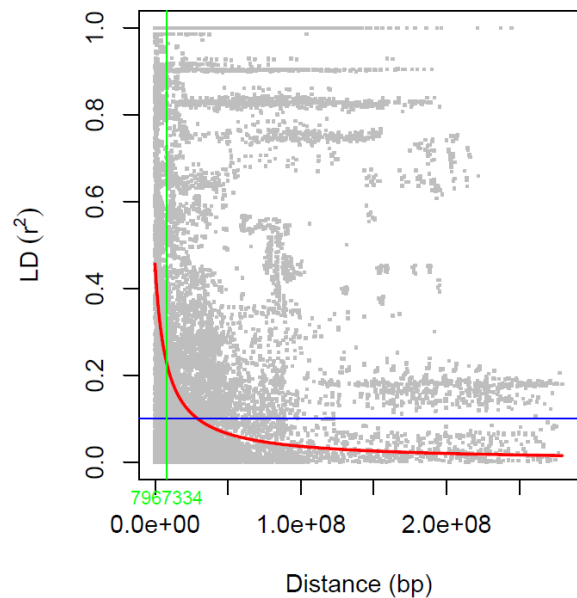**6B**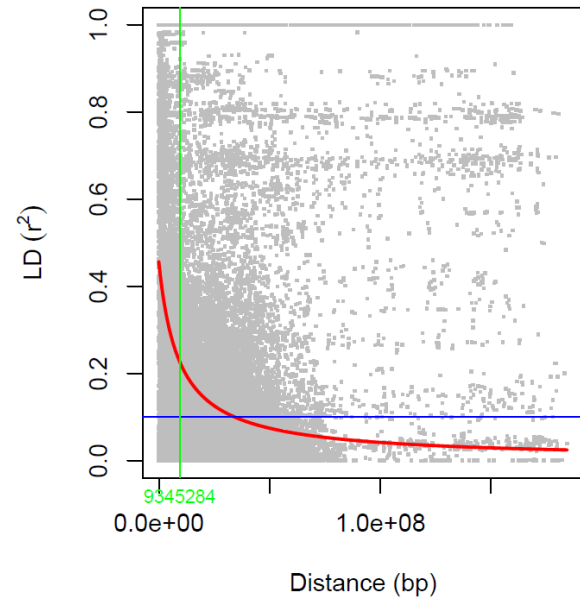**6D**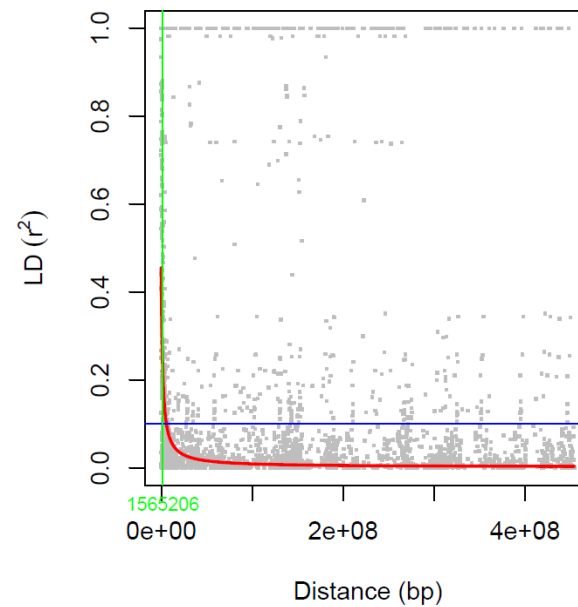**7A**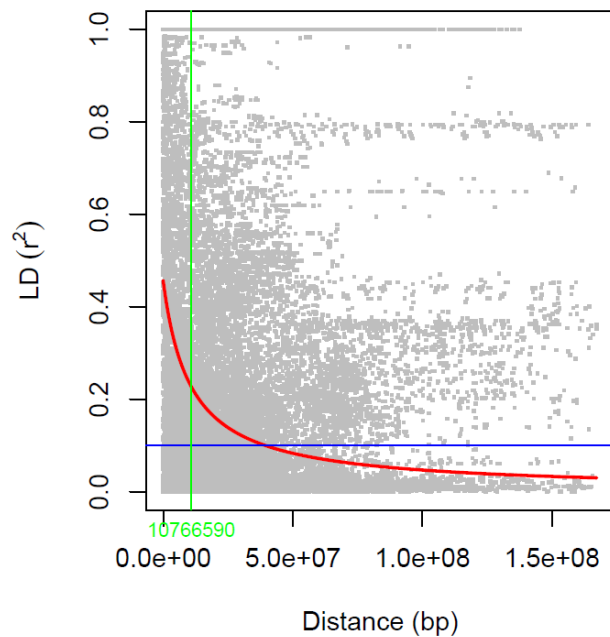**7B**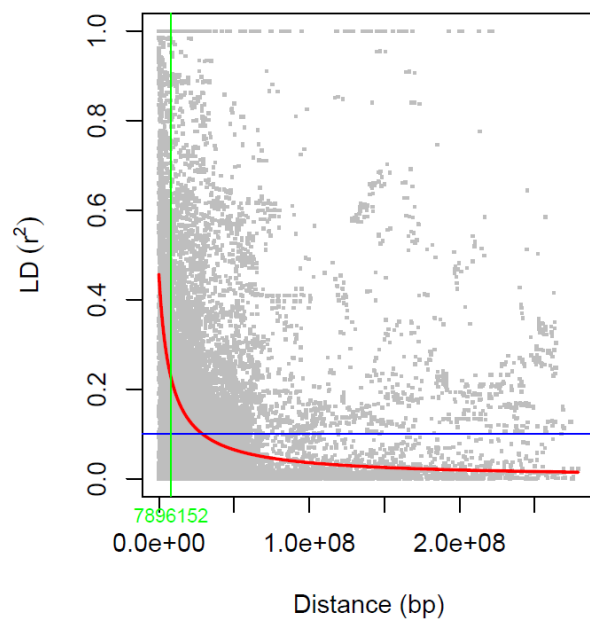**7D**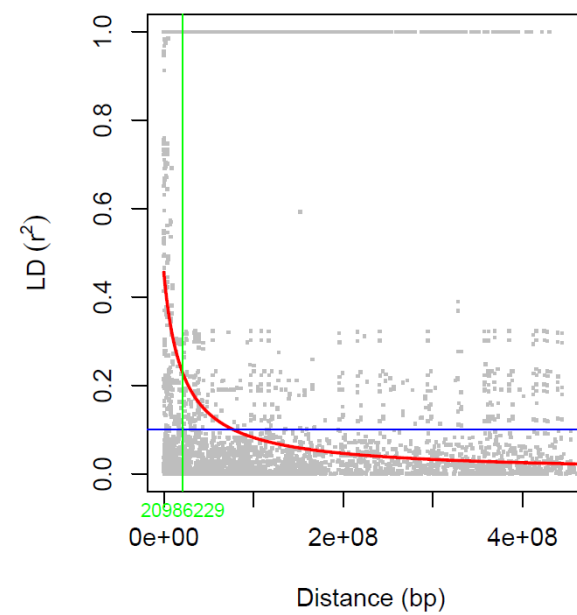

Supplement: Supplementary file 1 [file plants-13-02623-s001.zip › Figure S2.pdf]
